# Retroperitoneal appendicitis within a posterior abdominal wall incisional hernia secondary to a right nephrectomy: a first incidence case report

**DOI:** 10.1093/jscr/rjag066

**Published:** 2026-02-12

**Authors:** Lithira R Walisinghe, Frana Katica Batistic, Michael C Auld

**Affiliations:** Department of General Surgery, Ipswich Hospital, Chelmsford Avenue, Ipswich, QLD, 4305, Australia; Department of General Surgery, Ipswich Hospital, Chelmsford Avenue, Ipswich, QLD, 4305, Australia; Department of General Surgery, Ipswich Hospital, Chelmsford Avenue, Ipswich, QLD, 4305, Australia

**Keywords:** acute appendicitis, retroperitoneal appendicitis, appendiceal herniation, nephrectomy, incisional hernia, mesh hernioplasty

## Abstract

Appendiceal herniation is rare and typically described in inguinal or femoral hernias. Retroperitoneal appendicitis within a nephrectomy incisional hernia has not previously been reported. A 65-year-old woman with a history of flank approach right nephrectomy presented with migratory flank-to-umbilical pain and raised inflammatory markers. Computed tomography demonstrated acute appendicitis herniating through a posterolateral abdominal wall defect at the prior nephrectomy site. She underwent open appendicectomy via the original flank incision, followed by single-stage hernia repair using biological mesh. Postoperative recovery was uncomplicated apart from a small conservatively managed seroma, and histopathology confirmed acute appendicitis. This case highlights a previously undescribed presentation of appendicitis and underscores the importance of individualized operative planning when considering concurrent hernioplasty in a potentially contaminated field.

## Introduction

Appendiceal involvement in hernias is rare but well described in specific contexts, including Amyand, De Garengeot, Spigelian, and umbilical hernias [1–4]. More unusual presentations have been reported, such as appendiceal herniation within a midline incisional hernia following open cholecystectomy, and retroperitoneal migration beneath the diaphragm after diaphragmatic hernia repair [5, 6]. While flank bulging and lumbar herniation are recognized long-term sequelae of nephrectomy, our literature search identified no prior reports of appendiceal herniation through a retroperitoneal defect following nephrectomy [7].

Management of hernias containing the appendix requires careful operative judgment, beginning with selection of surgical approach. Although laparoscopic resection of a retroperitoneal appendix has been described, many cases require conversion to an open approach due to technical difficulty, or utilize modified open techniques such as a utilizing pre-existing surgical scars or subcostal incisions [5, 6, 8, 9].

Equally important is determining the optimal method of abdominal wall reconstruction. Acute appendicitis within a hernial sac results from extraluminal compression at the narrow hernia neck, leading to vascular compromise, ischaemia, and bacterial invasion [10]. Perforation within a hernia significantly increases the risk of peritonitis, creating uncertainty regarding the safety of prosthetic materials in a contaminated field [10]. Although primary suture repair may be appropriate when defect size permits, mesh-based repair is generally considered superior [11, 12]. When mesh is used, selection requires balancing the reduced infection risk of biologic materials against the lower recurrence rates associated with synthetic mesh [4, 5, 13, 14].

Whether appendicectomy and hernia repair should be staged remains debated. Both simultaneous repair and delayed reconstruction have been reported, with delay often intended to reduce infection and mesh contamination [1, 3–5, 13].

We present a first documented case of retroperitoneal appendicitis within an incisional hernia following nephrectomy in a 65-year-old female. Managed with open appendicectomy and biological mesh repair, this case highlights the challenges of altered anatomy, surgical approach, and abdominal wall reconstruction in a potentially contaminated field, underscoring the need for individualized operative decision-making in a consensus scarce situation.

## Case report

A 65-year-old female presented with a 4-day history of right flank pain migrating to her umbilicus. The patient’s surgical history was significant for a lateral flank approach right nephrectomy 25 years prior, due to recurrent infections, abscesses, and an operative tract secondary to an initial staghorn calculus. Her pain was focally reproducible upon palpation around the nephrectomy scar. Laboratory investigations demonstrated mildly elevated inflammatory markers (white cell count 11 × 10^9^/L, C-reactive protein level 65), and her vitals were stable with no fevers. Additional surgical history included an open cholecystectomy 15 years prior, and medical history included an unprovoked deep vein thrombus 7 years prior, hypothyroidism, hypertension, gout and obesity.

**Figure 1:**
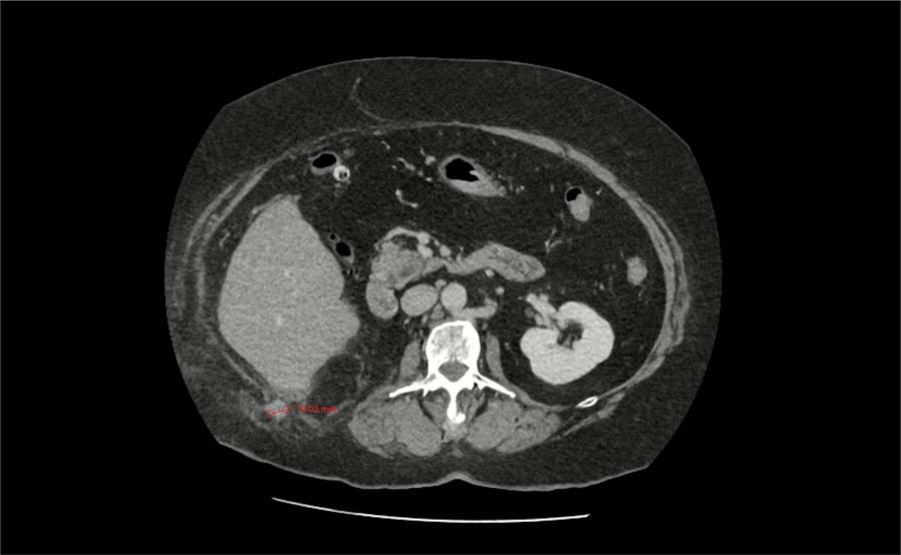
CT axial section showing a pathologically thickened appendix of 12 mm diameter (red measurement) projecting through a right posterolateral abdominal wall defect. There is also notable inflammatory fat stranding, with no evidence of perforation.

**Figure 2:**
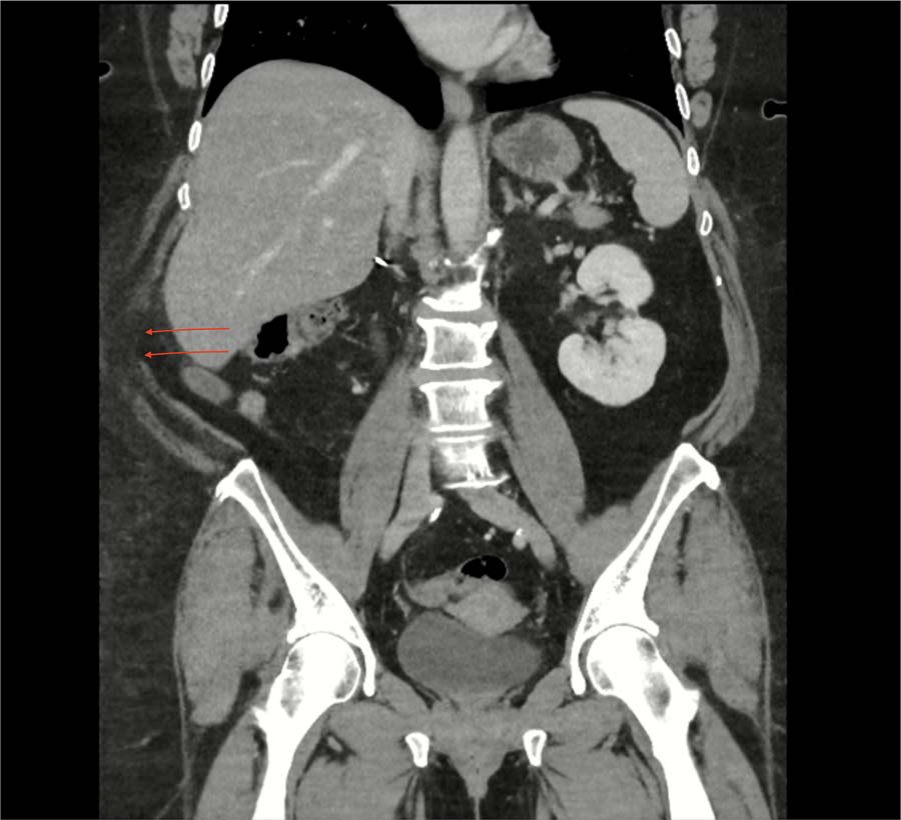
CT coronal section highlighting notable right posterolateral abdominal wall defect (arrows).

**Figure 3:**
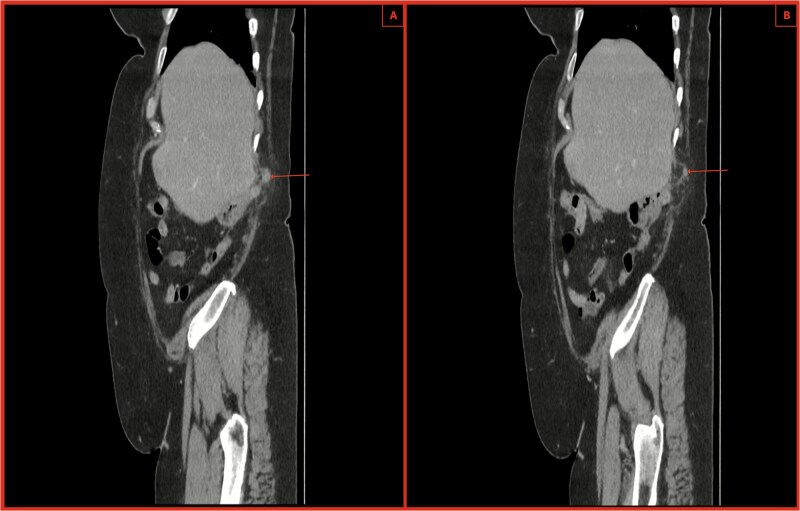
(A, B) CT sagittal sections with notable posterior abdominal wall hernial defect and appendix within hernia highlighted by arrows. There is also notable inflammatory fat stranding, with no evidence of perforation.

An urgent computed tomography (CT)-abdomen/pelvis highlighted an inflamed appendix extending into a small hernial defect in the right posterolateral abdominal wall (Figs 1–3). Thus, diagnosing retroperitoneal acute appendicitis within a right nephrectomy incisional hernia. The patient was started on intravenous Ceftriaxone and Metronidazole prior to deciding upon an open appendicectomy and repair of the incisional hernia using biological mesh.

**Figure 4:**
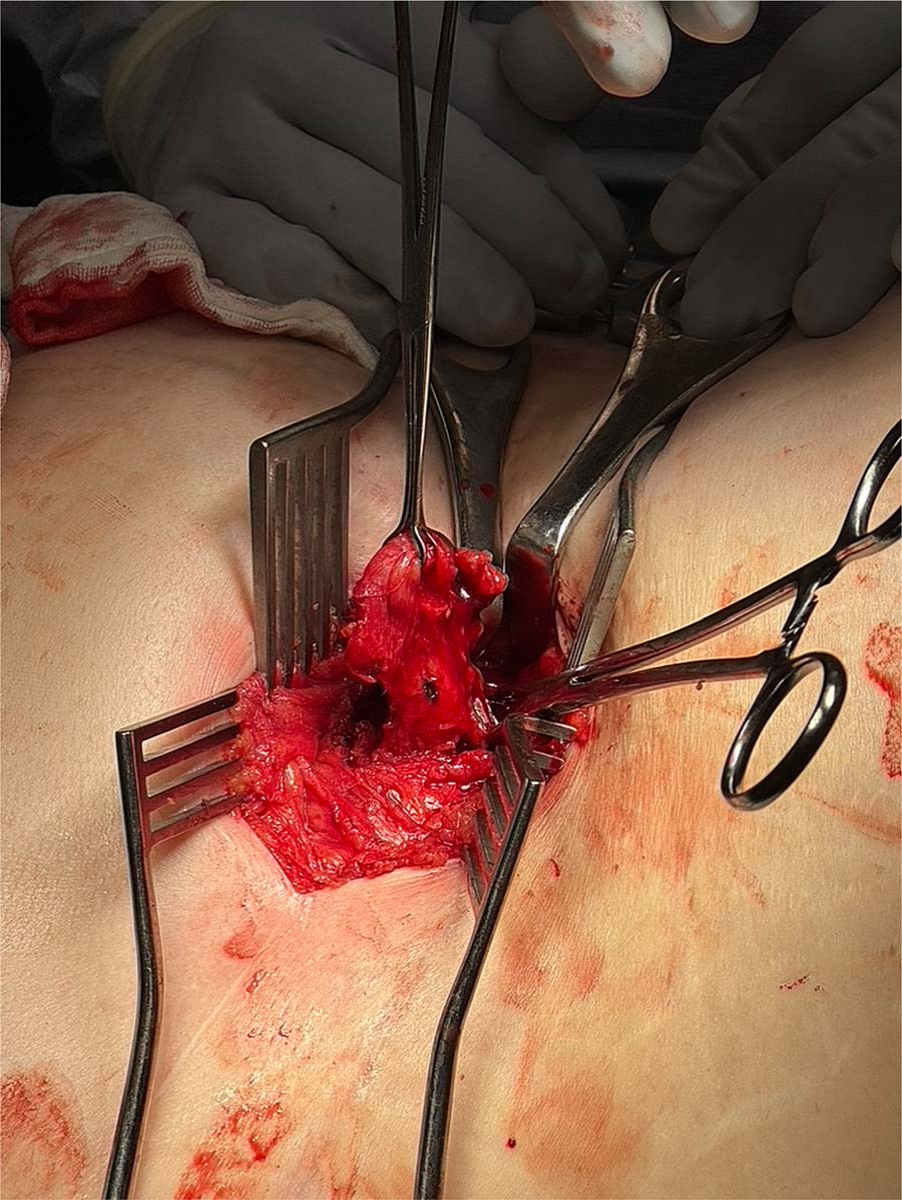
Intraoperative image showing entry into the posterior abdominal wall hernia using the same right nephrectomy scar. The peritoneum is opened and appendix is isolated.

**Figure 5:**
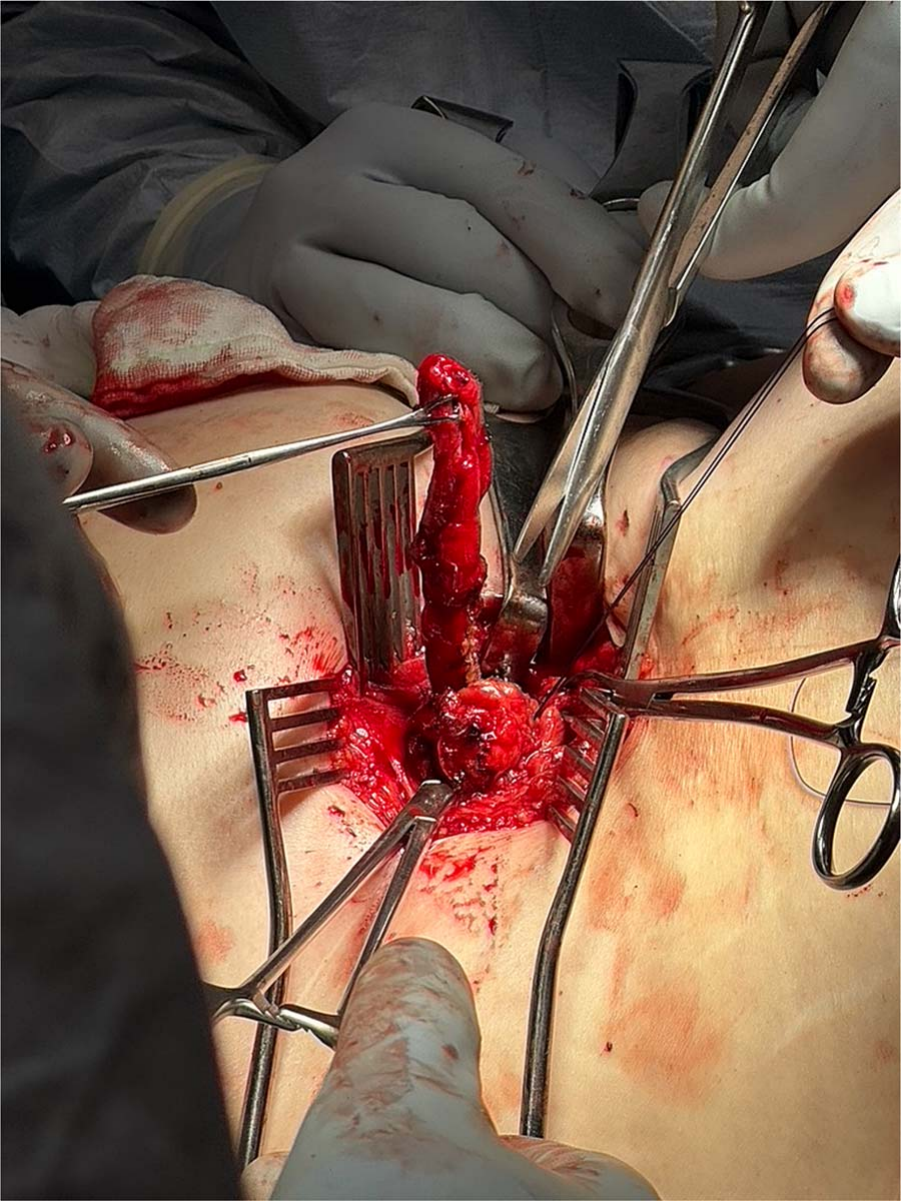
Intraoperative image showing isolated appendix with base being tied off with 0-Vicryl.

**Figure 6:**
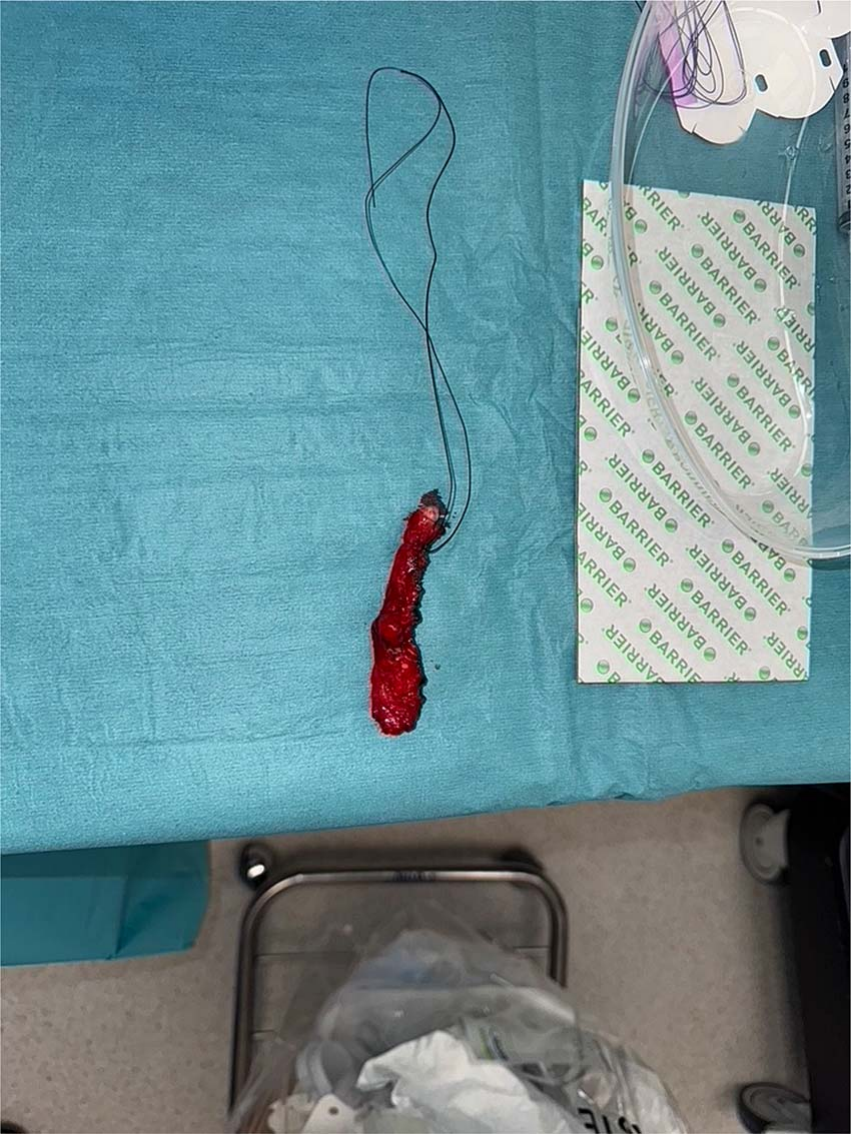
Intraoperative image showing excised appendix with no signs of perforation.

**Figure 7:**
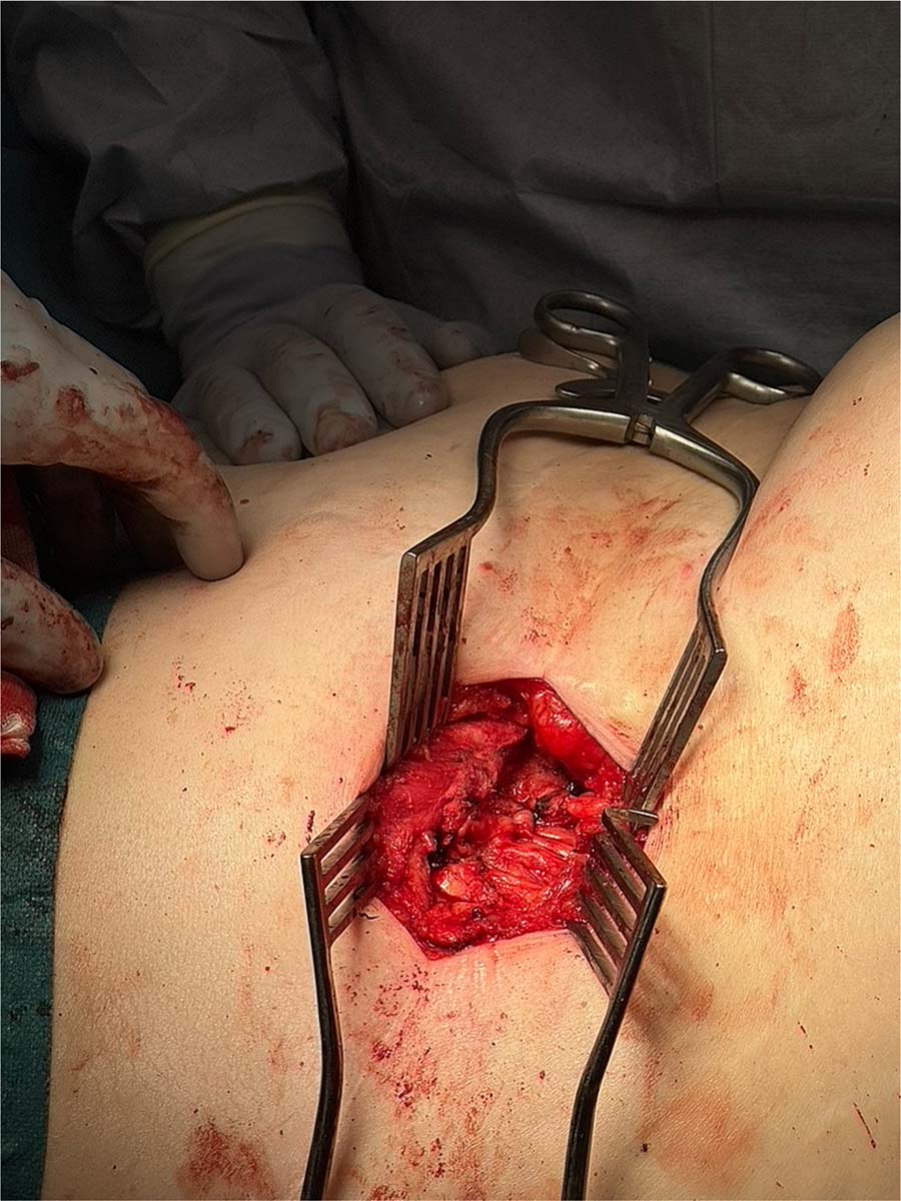
Intraoperative image showing external oblique fascial plane closed with 0-PDS, prior to insertion of Phasix biological mesh.

**Figure 8:**
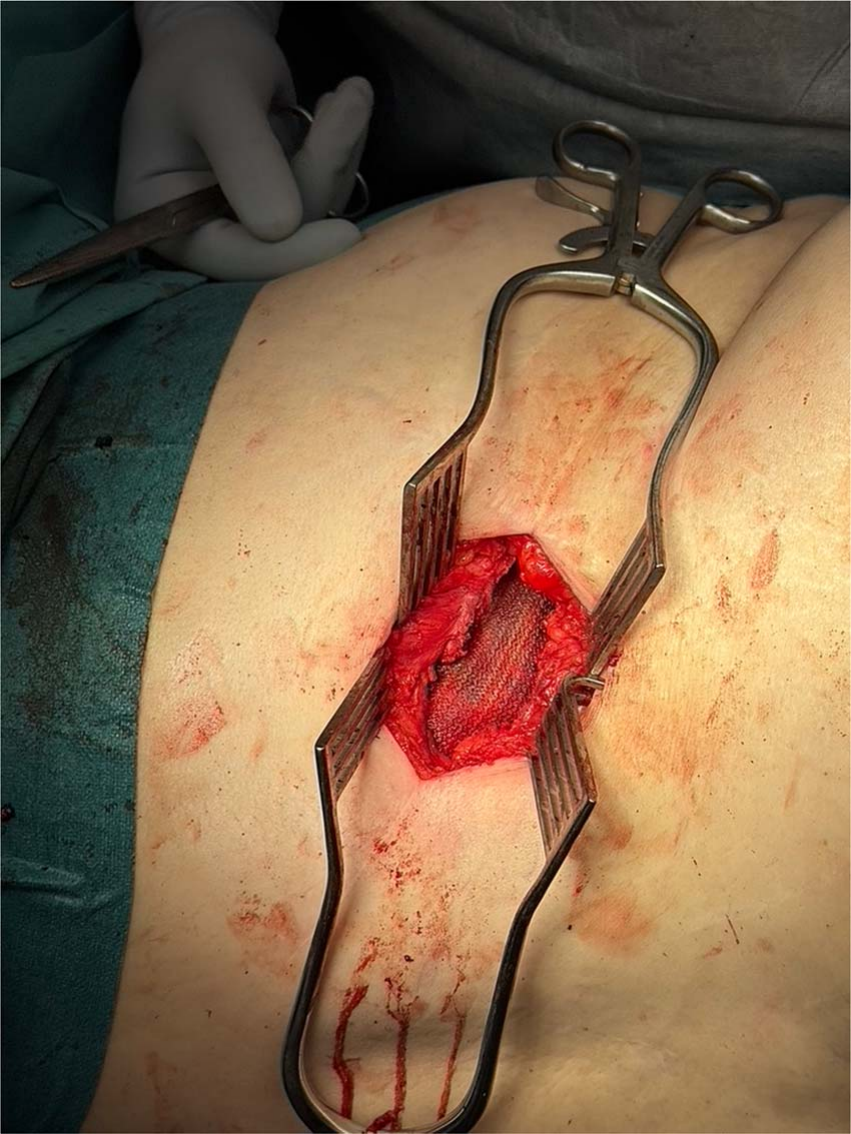
Intraoperative image showing Phasix biological mesh (in view) placed onlay over repaired hernial defect.

The operation utilized a left lateral position and posterior approach using the same right nephrectomy scar. A 3-cm posterior abdominal wall hernia was identified, hernial sac defined, and peritoneum opened (Fig. 4). The appendix showed no evidence of perforation, and the base was tied off using 0-vicryl, then excised (Figs 5 and 6). The external oblique fascial plane was defined and identified to be healthy, then closed with 0 Polydiaxanone (PDS) suture and washed (Fig. 7). A Phasix® biological mesh was placed onlay and anchored with 2–0 Prolene to the subcostal and external oblique fascia (Fig. 8). Postoperatively, the patient received one additional day of intravenous antibiotics and was discharged with an abdominal binder and 5-day course of oral Augmentin-Duo Forte.

The patient developed a small non-infected subcutaneous seroma at three weeks, which was managed conservatively without antibiotics. At the 4-week follow up she was progressing well, and histopathology confirmed acute appendicitis.

## Discussion

This case is novel due to its unique surgical challenges, requiring multiple decisions to be made based on a case-by-case basis in the absence of robust evidence. To our knowledge, this represents the first documented case of retroperitoneal appendicitis within a nephrectomy incisional hernia. While the acute pathology of appendicitis appropriately takes precedence, the concurrent incisional hernia also necessitates careful consideration.

A key initial decision concerns whether to perform a delayed or single-staged hernia repair in the setting of a potentially contaminated surgical field. Delayed repair will likely reduce the risk of wound and prosthetic infection following index operation [13]. However, this must be weighed against the risk of progressive enlargement of the hernial defect and the possibility of bowel incarceration [13]. A staged approach also inevitably exposes the patient to risks pertaining to a second operation, repeat general anesthesia and also risks associated with patient-specific comorbidities.

If a single-stage repair is chosen, the surgeon must then decide between mesh and non-mesh repair. Mesh repair has long been established as superior to suture repair, even for small hernias, with some studies demonstrating a 50%–75% reduction in recurrence rates [11, 12]. Nonetheless, mesh implantation carries an increased infection risk in contaminated or potentially contaminated fields, and therefore sterility of the site must be carefully assessed [12].

The final consideration is the choice between biological and synthetic mesh. While there is some evidence showing lower recurrence rates with heavyweight synthetic meshes, there is substantial evidence indicating higher rates of surgical site and mesh infection when compared with biological meshes [14, 15]. Consequently, mesh selection should be guided by several factors including the size of the hernial defect, anticipated tissue tension, patient comorbidities, and, most critically, the risk of infection [12, 14, 15].

In our case, there was no radiological or intraoperative evidence of appendiceal perforation, and the surgical field was deemed clean. A single-stage repair post appendicectomy was therefore undertaken to minimize the risk of future bowel incarceration and avoid a second operation, particularly given the patient’s age and comorbidities. A biological mesh was selected, balancing recurrence risk and infection potential, given that the defect size was only 3 cm and due to the inability to guarantee absolute sterility.

This case highlights the operative implications in managing concurrent diagnoses of an acute surgical pathology that is appendicitis within a post-nephrectomy retroperitoneal incisional hernia.

## Conclusion

This case demonstrates the successful management of a previously unreported presentation of retroperitoneal appendicitis within a nephrectomy incisional hernia. In the absence of established guidelines and diagnosis-specific evidence, management required careful, individualized decision-making regarding operative approach and abdominal wall reconstruction, informed by broader hernia and appendicitis literature.
